# The role of cadre in the community on diabetic retinopathy management and its challenges in low-middle income countries: a scoping review

**DOI:** 10.1186/s12889-024-17652-5

**Published:** 2024-01-15

**Authors:** Irma Suwandi Sadikin, Yeni Dwi Lestari, Andi Arus Victor

**Affiliations:** 1 Residency Program in Ophthalmology, Faculty of Medicine Universitas Indonesia, Cipto Mangunkusumo General Hospital, Jakarta, Indonesia; 2 Ophthalmology Department, Faculty of Medicine Universitas Indonesia, Cipto Mangunkusumo General Hospital, Jakarta, Indonesia

**Keywords:** Diabetic retinopathy, Diabetic eye screening programs, Cadre, Eye health promotion

## Abstract

**Introduction:**

Diabetes is a serious public health problem, with low- and middle-income countries (LMICs) bearing over 80% of the burden. Diabetic retinopathy (DR) is one of the most prevalent diabetic microvascular problems, and early diagnosis through eye screening programs for people with diabetes is critical to prevent vision impairment and blindness. Community-based treatments, including non-physician cadres have been recommended to enhance DR care.

**Methods:**

The review protocol was determined and scoping review was conducted.The population, concept, and context were “cadre”, “role of cadre in the management of DR”, and LMICs”. Data were collected from databases and searches, including grey literature.

**Results:**

Cadre can motivate people to attend a diabetic eye screening event when the rate of eye examinations is about six times higher than before the start of the intervention. Health education is a possible area for task sharing, and the cadre reported could also perform the task of vision testing. The cadre could be a good supporter and a good reminder for society. However, several challenges have been faced in this study and inadequate infrastructure is the foremost challenge found in this study. Other challenges encountered in the studies include poverty, lack of community awareness, trust issues, and low education levels contributing to poor health.

**Conclusion:**

The current study highlighted significant gaps in the literature, which focus on the role of cadre as a community-based intervention in managing DR in LMICs. Further research is needed to develop evidence to support cost-effective screening services and cadre-related policy development in LMICs.

**Supplementary Information:**

The online version contains supplementary material available at 10.1186/s12889-024-17652-5.

## Background

Diabetic retinopathy (DR) is a microvascular disorder resulting from prolonged exposure to diabetes mellitus. It represents a grave complication of diabetes and is the foremost cause of blindness globally. As per the estimates provided by the International Diabetes Federation (IDF), the worldwide population suffering from diabetes mellitus (DM) was 463 million in 2019, and it is projected to increase to 700 million by 2045 [1]. This upsurge in diabetes prevalence is accompanied by a heightened risk of DR and its associated complications. The Global Burden of Disease Study identifies DR as the fifth most common cause of blindness and moderate to severe vision impairment in adults aged 50 and above and also the main cause of vision impairment in adults of productive ages [2]. Diabetic maculopathy and complications of proliferative DR, such as vitreous hemorrhage, tractional retinal detachment, and neovascular glaucoma, account for the majority of vision loss [3].

Furthermore, the increasing number of diabetics also increases the incidence of DR. According to the American Academy of Ophthalmology's most recent epidemiological data, the number of adults worldwide with DR is estimated to be 103.12 million, with a projected increase to 160.50 million (55.6%) by 2045 [4]. In low- and middle-income (LMICs) countries, especially in Indonesia, the DR population was 30.7% among diabetics populations, with 7.6% suffering from vision-threatening DR. Ages 50 and up, diabetes duration of five to ten years, and more than ten years, and postprandial blood glucose of 200 mg/dl or higher were all associated with a higher prevalence of any DR [5, 6]. As the number of diabetes patients grows, so is the demand for ophthalmic care patients and medical specializations (e.g., exams, screening, treatments, and ophthalmologists).

The screening target for DR cases is expected to reach 80% in 2030, as determined by the WHO [7, 8]. Therefore, a cost-effective and efficient program is needed to achieve this target. The development of optimal screening programs using accessible ophthalmic infrastructure resources is prominent, considering it has been proven more cost-effective than no or opportunistic screening [9, 10]. Furthermore, ophthalmologists have been occupied with a massive growing population of people with diabetes (PwD); it is not realistic to rely on ophthalmologists to examine the whole population, so ophthalmologists need help from community representatives to achieve the target of health services primarily to remote areas.

Another issue regarding the screening process is awareness regarding DR as a complication of diabetes due to the lack of literature in society. Mostly, diabetic patients didn't seek any eye examination until they developed symptoms. As a result, patients are frequently identified with DR in a severe, vision-threatening stage, making treatment challenging [11, 12]. Nevertheless, cost-effectiveness screening necessitates high coverage, which is problematic in LMICs, where accessible eye health services are concentrated in cities and access to them from remote and rural populations would be difficult [13].

In addition, the insufficiency of ophthalmologists in LMICs, particularly in Indonesia, is a significant barrier to tackling the higher prevalence of DR. The ratio of ophthalmologist density to population is 1:155,618; it should be 1:250,000, as targeted by the Ministry of Health in "Peta Jalan Penanggulangan Gangguan Penglihatan di Indonesia Tahun 2017–2030" [14]. The uneven distribution of ophthalmologists also exacerbates the lack of numbers, so people in remote villages struggle to access adequate eye care services [14]. Furthermore, screening at the primary level using non-medical people is suggested by WHO, which would help cover a larger population. In response to anticipated interest among policymakers in many countries in resolving the health worker shortage, the World Health Organization (WHO) supported task shifting by cadre as a community-based intervention [15]. Cadrs are non-medical individuals who undergo specific medical training to serve in designated roles within the community [16]. They encompass various titles that are adapted to specific countries, such as social workers, lady health workers, community health workers, and village health workers [11, 17]. The World Health Organization (WHO) defines task shifting as the delegation of responsibilities either to existing healthcare professionals with less training and credentials or to newly established cadres who receive competency-based training for particular activities [18].

Cadre should be part of the healthcare system by helping doctors and nurses with eye screening and health promotion tasks [16, 19]. In this way, they are seen as 'another pair of hands', as they contribute to providing care to underserved communities and increasing the health system's capacity to deal with financial and human shortages in a resource-poor situation [18]. Cadres have been regarded as social and cultural intermediaries and supporters, enhancing the link between the current health system and the community [11]. Therefore, their job should be facilitating community engagement and taking the necessary steps to overcome the social and cultural barriers contributing to poor health [20].

Community-based interventions involving non-physician cadres have been proposed to improve the management of DR. Screening at the primary level performed by cadre would help cover a larger population. In addition, community-based interventions can reactivate the healthcare pyramid to achieve universal coverage. However, the extent and nature of the literature on the role of cadres in the community on DR management, and the challenges associated with implementing such interventions, are poorly understood. This scoping review aims to map the available literature on the role of cadre in the community on DR management, with a particular focus on the challenges associated with implementing such interventions. By identifying gaps in the literature and highlighting areas for further research, this scoping review can inform the development of community-based interventions to improve the management of DR in LMICs.

## Methods

### Literature searching and concept scoping review

A scoping review is conducted using the Arksey and O'Malley methodological framework. Preferred Reporting Items for Systematic Reviews and Meta-Analyses Extension for Scoping Reviews (PRISMA-ScR) criteria also were followed in conducting and reporting the scoping review [21, 22].

The 'PCC' mnemonic is recommended as a guide to constructing a clear and meaningful title for a scoping review. The PCC mnemonic stands for population, concept, and context. The population is an essential characteristic of participants, and characteristics should be detailed, including age and other qualifying criteria that make them appropriate for the objectives of the scoping review and the review question. Next, a concept should be clearly articulated to guide the scope and breadth of the inquiry. This may include details on elements in a formal systematic review, such as the 'interventions' and/or 'phenomena of interest' and/or 'outcomes.' Last, context may include cultural factors such as geographic location and/or specific racial or gender-based interests. In some cases, context may also encompass details about the particular setting. The population, concept, and context were “cadre”, “role of cadre in the management of DR”, and LMICs”, respectively.

### Data sources and search strategy

This scoping review is as comprehensive as possible without a time limit to obtain the data. The primary sources used were PUBMED/MEDLINE, Embase, and the Cochrane Database of Systematic Reviews (CDSR), and the Cochrane Central Register of Controlled Trials (CENTRAL) in the Cochrane Library. Manual searching and grey literature were obtained from reference lists of included articles. Details of keywords with or without MeSH terms that have been used are listed in Supplementary file 1. In addition, a manual search was also performed by checking the reference lists of all the retrieved studies to identify studies not yet included in computerized databases.

### Study selection

The eligibilities of the study are (1) provide non-medical and non-paramedic personnel (cadre) despite of the term used in their research; (2) offer the explanation of the cadre’s role; (3) involve cadre in screening for DR detection: assessment of the target population for the presence of DR by individuals other than ophthalmologists through history-taking, (4) be conducted in LMICs to generate evidence to inform the development of national- or subnational-level DR screening and treatment programs, (5) be published in English. However, titles and abstracts that did not meet the eligibility criteria were excluded, and full-text articles were retrieved for those that did meet the criteria. The selected studies were rated using the Oxford Center of Evidence-based Medicine Levels of Evidence [23].

### Data extraction

Data were gathered from each study that met the inclusion and exclusion criteria. The author(s), year of publication, study location in low-middle income countries, study populations (cadre/community health workers/lady health workers/village health worker), aims of the study, methodology, characteristics of cadre (including age, numbers, the scope of the task, and training process of cadre), the role of cadre in society, society response towards cadres, and challenges/ barrier.

### Operational definition

The operational definitions of the terminology used for this study is summarized in Supplementary file 2 [6, 18–23].

## Results

### Search results

**Fig. 1:**
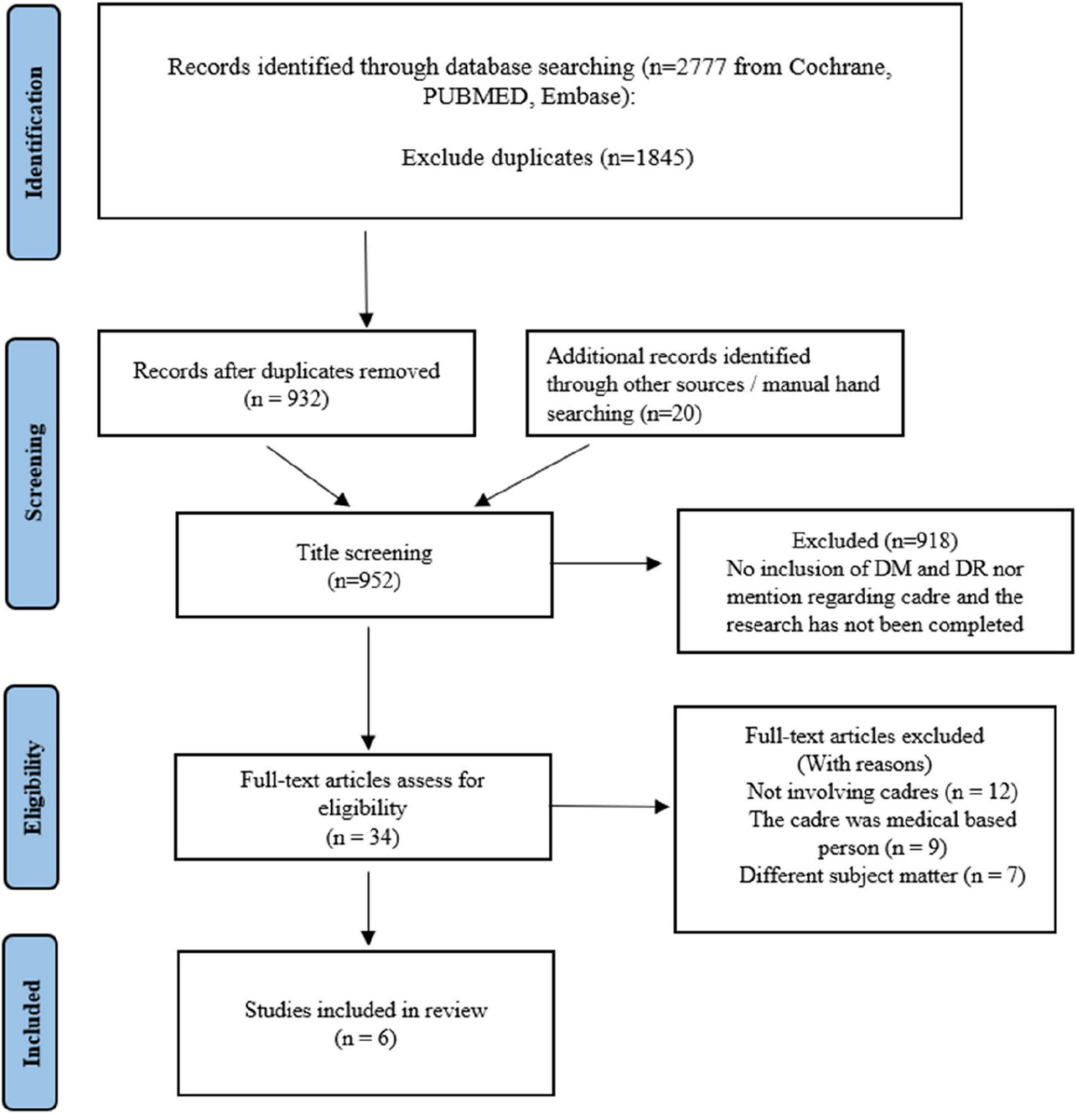
Prisma flow chart of eligible studies for scoping review

The search yielded 2777 articles, with an extra fifteen entries discovered from other sources (websites and bibliographies). Then, Mendeley software removed duplicates (1845/2777; 66%). After that, additional records were identified through other sources or manual hand searching for 20 articles. Next, 918 articles were excluded because they did not include DM, DR, or cadre in the title, and many of the articles had not been completed for the study. Finally, 34 articles were assessed for eligibility with full text. As a result, six articles meet our inclusion criteria. Figure 1 summarizes the process of searching and recruiting for this study.

### General characteristics of included studies

**Fig. 2:**
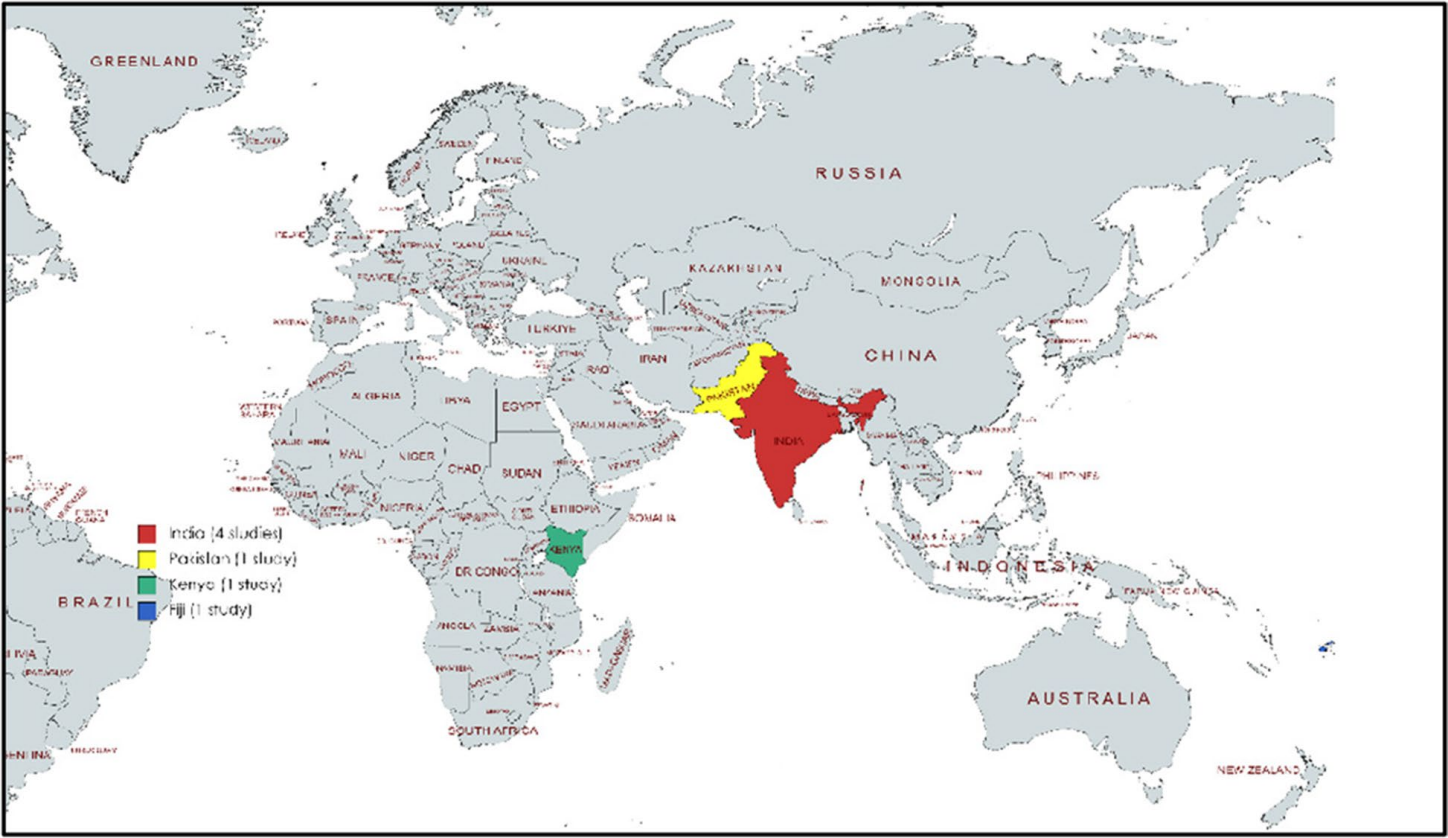
Map of studies

The included research studies and reports were published between 2005 and 2020 and comprised 6 published studies including interventional studies (*n* = 4) and qualitative studies (*n* = 2). All included documents were in English, where most studies were interventional studies, including one randomized clinical trial and two qualitative studies with II-III levels of evidence. All countries in our study are from LMICs, with India being the dominant country. Other countries were Fiji, Pakistan, and Kenya. The data about the distribution of our study is shown in Fig. 2.

### Cadre terms and the purpose

This study uses a variety of cadre words. Cadre terms are tailored to the countries and conditions handled by the cadre. Rani et al., for instance, refer to them as cadre social workers since they are voluntarily involved in the social community to assist medical workers in connecting with the community [16]. Fiji, on the other hand, defined cadre as community health workers (CHW) or village health workers (VHW), which have the same meaning: health care providers who live in the community they serve and receive lower levels of formal education and training than professional health care workers such as nurses and doctors. While in Pakistan, the cadre is known as lady health workers (LHW) who must have at least 8 years of schooling, be recommended by their community, and undergo extensive training to become a lady health worker [11, 24]. Every program health worker is allocated to a specific government health institution, where they receive training, a limited subsistence allowance, and medical supplies. Also, provincial and district coordinators supervise the Lady Health Worker Program which holds quarterly review meetings and provides analytical input on health records from LHW [25, 26]. In India, Cadres are addressed as ASHA, or accredited social health activists, women village residents who are married, widowed, or divorced, preferably between the ages of 25 and 45. They are selected and trained to act as a mediator between the community and the public health system [27].

The majority of cadres involved in this research shares several primary tasks. These individuals play a crucial role in serving local communities, particularly those residing in remote areas and historically marginalized populations. Firstly, they aim to raise awareness about diabetes and diabetic retinopathy within society. This awareness encourages individuals to willingly undergo screenings for early detection and prompt treatment of sight-threatening diabetic retinopathy. Secondly, the screening process helps in initiating the referral process, categorizing patients based on their need for further evaluation, direct treatment, and even surgery. While some cadres have relatively straightforward responsibilities, others, like India's ASHAs, have more complex duties. ASHAs, for instance, are required to familiarize themselves with the health status of villagers. They visit every family and conduct sample surveys of the village's population to assess their health [27]. Additionally, ASHAs perform basic tasks such as glucose screening and rudimentary visual testing, which significantly assist medical officers in their duties [16, 17]. Task shifting proves to be highly beneficial in redistributing the duties and responsibilities of ophthalmologists within the community. These cadres play a crucial role in maximizing the screening of eye problems at the grassroots level. The purpose and diversity of cadres, as well as their roles, will be summarized in Table 1 in the comprehensive review of related studies.

**Table 1:** Cadre terms of studies and their purpose

No	Author	Year	Country	Methods	Purpose	Cadre terms	Evidence based level
1	Rani et al	2005	India	Two arm randomized controlled trials (Pilot study)	1. To create awareness about diabetes and diabetic retinopathy in the general population in Indian rural areas 2. To conduct diabetic screening camps for early detection and prompt treatment of sight threatening diabetic retinopathy 3. To train general ophthalmologists and general physicians in diagnostic techniques to identify patients at risk of developing diabetic retinopathy 4. To perform relevant biochemical and genetic investigations to discover the risk factors associated with the development of diabetic retinopathy	Social workers	II
2	Singh S et al	2020	India	Interventional study	1. Primary outcome: uptake of DR screening at facilities with and without health education 2. Secondary outcome: uptake of screening by type of facility (CHC, PHC) and also transport was provided	Village Level Health Workers (VHWs) Social health activist (ASHA)	II
3	Mwangi et al	2020	Kenya	Intervention Study	To test the effectiveness of peer support in increasing the uptake of retinal examination among members of diabetes support groups	Peer supports of diabetes support groups (DSG)	II
4	Ram S et al	2022	Fiji	Qualitative study	To explore the impact of the training on CHWs’ knowledge of DR and its influence on their referral processes, identify challenges faced by CHWs in referrals for DR screening, and their integration within the health system	Community health workers (CHWs)	III
5	Shah et al	2018	Pakistan	Qualitative study	To develop a checklist of tasks, based on the opinion of eye care professionals, to be possibly shared by optometrists and mid-level eye and health care workers in eye care delivery for people with diabetes	Lady health workers (LHW)	III
6	Chariwala et al	2020	India	Non-randomized controlled trial	The impact of the ASHA volunteers for DR screening promotion with and without incentives	Accredited Social Health Activists (ASHAs)	III

*ASHA *Accredited social health activist, *CHC *Community health centers, *CHWs *Community health workers, *DR *Diabetic retinopathy, *DSG *Diabetes support groups, *LHW *Lady health worker, *PHC *Primary health care

### Study results

#### The importance of cadres in local community

DM is increasing around the world. The IDF estimates that the number of people with DM will reach 700 million in 2045 [1]. The increasing number of diabetics also increases the incidence of DR [4]. In fact, DR is an important cause of vision impairment and blindness [2]. On the other hand, there is strong evidence that good control of DM and associated systemic conditions reduces the incidence of sight-threatening retinopathy and improves prognosis after standard treatment of DR [7].

However, human resources are a constraint in LMICs. An acute shortage of retina specialists causes overwhelming work to handle this issue [14, 28]. Health worker shortages can impede access to quality healthcare services, and the impact is exacerbated if such shortages are followed by unequal worker distribution [29, 30]. Screening at the primary level using non-ophthalmic, trained technicians would help cover a larger population. In response to anticipated interest among policymakers in many countries in resolving the health worker shortage, the WHO supported task shifting by cadre as a community-based intervention [15].

Cadre should be part of the healthcare system by helping doctors and nurses with eye screening and health promotion tasks [16, 19]. In this way, they are seen as 'another pair of hands', as they contribute to providing care to underserved communities and increasing the health system's capacity to deal with financial and human shortages in a resource-poor situation [18]. Cadres have been regarded as social and cultural intermediaries and supporters, enhancing the link between the current health system and the community [11]. Therefore, their job should be facilitating community engagement and taking the necessary steps to overcome the social and cultural barriers contributing to poor health [20].

#### Characteristic cadres

The average age of the cadre in this study was > 18 years old, but one study included ages from 25 to 65 years old. The setting of this study took place in a rural area and included a community. All the cadres in this study were trained to perform their community tasks. The training included health education, such as screening, and inviting the community to participate in the screening event and eye examination. In addition, they also supported the community in obtaining treatment and served as a reminder to control the patient's treatment and help refer the patient to a higher health facility for further examination by an ophthalmologist. One study by Shah et al. assessed that cadres could perform simple vision checks for patients. This skill is beneficial for task shifting, which can be utilized as an initial vision screening stage. The role of this cadre can optimize the screening of eye issues at the most basic level by dividing the responsibilities and functions of ophthalmologists in the community with task-shifting.

Furthermore, educational media used by cadres in educating the community can be in the form of leaflets, posters, education sheets, peer-to-peer discussions, annual meetings, and mobile phone groups tailored to the needs and events provided to patients. The media is considered helpful in conducting health promotion in the community. The use of the local language is also the most critical thing in carrying out health promotion in the community in the area; therefore, almost all cadres must be able to speak and use local languages to participate in the research project [31, 32]. Information regarding the cadre’s characteristics will be summarized in Table 2.

**Table 2:** Table of cadre characteristic

No	Author	Year	Country	Cadre Characteristic					
				**Age**	**Numbers of cadre**	**Setting**	**Did they receive training?**	**Media used by cadre for education**	**Scope of the task**
1	Rani et al	2005	India	NA	10	Rural	Yes	Poster, leaflet, oral explanation	- Health education - Screening
2	Singh S et al	2020	India		-	Rural	Yes	Poster, leaflet, oral explanation	- Health education - Screening - Facilitate community to attend for screening
3	Mwangi et al	2020	Kenya	> 18 y.o	2 peers educators in each group (1 male and 1 female)	Rural	Yes	- Peer to peer (meeting—the meetings are held in the morning, starting between 8 and 9 am and last 2–3 h) - Group discussion - Media communication using mobile phone	- Health education and health promotion - Screening - Reminders society to attend screening and health education and promotion - Supporters
4	Ram S et al	2022	Fiji	25–65 y.o	14	Community	Yes	Peer to peer by oral explanation	- Health education and promotion - Screening - Referrals
5	Shah et al	2018	Pakistan	NA	25	Community	No	-	- Health education - Vision testing
6	Chariwala et al	2020	India	NA	55	Rural	Yes	Printed IEC materials in local language	- Health education - Screening - Referrals to health care (Paid vs unpaid)

*IEC* Information, education, and communication

#### The requirement to be cadre

All cadres in this study were given an orientation to the anatomy and physiology of the eye, diabetes and diabetic DR. They also have training regarding peer supporter training, a retinal screening, and communication [16]. Rani et al. in his randomized study conducted in India, stated that all cadre underwent intensive training for one week, eight hours a day. This training is to prepare them for the 2 most prominent events, which are World Diabetes Day and World Sight Day, to encourage society to attend the camp for screening [16].

Afterward, all ages cadre recruited in this study ranged between 18 and 65 years old. Following this, older cadres over 50 are still included because they have voluntarily served in the community for more than 15 years, so they are considered to have a good understanding of community conditions [33]. According to the interventional studies by Singh et al. in India and Mwangi et al. in Kenya, and also a non-randomized control study by Chariwala et al. in India, they employ cadres with experience in handling diabetic care and training and are already performing several health promotion activities [11, 31, 32].

#### The role of cadres in local community

From this study, a good-quality cadre from their training and selective selection process could create societal awareness. According to Ram et al., they conducted a survey of the cadre evaluating the impact of training. They discovered that the cadre thought that the activity had increased their knowledge of diabetes, its signs and symptoms, prevention, care and management, and DR as a diabetic complication, and also improved their communication skills [34]. As a result, people are motivated to attend the screening event because of the cadre’s motivation. In addition, the uptake numbers for screening were increased to achieve effective diabetic retinopathy screening [33].

In addition, the cadre could also help health workers remind society members who have high-risk diabetes to have regular screenings every month by telephone. They also collaborate with a primary health care worker to build a recall system to monitor and follow up on treated patients with diabetes and DR. The other activities of the cadre in society are making group health talks as peer supporters, informal discussions among PwD, planning for advocacy, and awareness-raising activities. Table 3 summarizes the role of cadres and the community's response to cadres.

**Table 3:** Role of cadre and community’s response to cadre

No	Author	Year	Country	Outcomes			
				**Cadre's quality**	**Role of cadre and societies behavior toward cadre**	**Information distribution effectiveness**	**Effectiveness of cadre in screening, referral, follow up DR**
1	Rani et al	2005	India	All cadres were given orientation to the anatomy and physiology of the eye, and also to diabetes and diabetic retinopathy	People are motivated to come to the screening event	Effective used media education such as pamphlets, leaflets, banners, lamp pole kiosks and audiovisual that were distributed one week before camps	- Increasing awareness before and after education - Can detect new diabetes mellitus case (4.5% that might have a risk for DR) - Community participation is the key to success for any awareness or screening model - The role of social worker was vital in the diabetic retinopathy-screening model for effective implementation; he or she served as a link between the community and diabetes healthcare professionals - An inbuilt recall system will monitor follow up of treated patients, as well as those patients who drop out of the program
2	Singh S et al	2020	India	- All cadre had been trained	- Good response - Enhance uptake the numbers of screening	Effective because of using local language	- Cadres could facilitate society's attendance at screenings in public health facilities or community health facilities using transportation services that were provided - Imparting health education to society - They are cost-effective because they have the potential to increase screening uptake in a relatively short period of time (3 months)
3	Mwangi et al	2020	Kenya	- All cadre had been trained - All cadres set aside time each month to meet with society	- Good response - Eighty percent of the members attend at least two thirds of the meetings annually	- Effective - Good monitoring - Good supporters	- The rate of incidence of eye exam was about 6 times higher in the intervention arm and striking uptake exam in the first two weeks of intervention - cadre can mobilized member to attend the screening - cost effective and efficient
4	Ram S et al	2022	Fiji	All cadres felt that the training had improved their knowledge of diabetes, its signs and symptoms, prevention and care/management, as well as on DR as one of the complications of diabetes	They are welcomed into the health facilities, feel respected, improved communication, and acknowledgement for their work made them feel part of the health system	All cadres reported sending a significant number of people to a health facility	Post-training, the cadre commenced referring not only the existing diabetes patients with vision loss or blindness for DR screening but also those who were 40 years of age or older and had other symptoms such as vision loss, blindness, or increased thirst, to visit a health facility for screening for diabetes
5	Shah et al	2018	Pakistan	All cadres had been given an explanation regarding the job that was standardized for optometrists and mid-level eye care workers	LHWs can play a role in identifying high risk people with diabetes along with their routine house to house visits for mother and child health care	Effective in education scope	- 88.5% cadre could perform task regarding education - 70% reported cadre could perform the task of vision testing
6	Chariwala et al	2020	India	- Experienced cadres (they have been performing several health promotion activities and specifically cataract case detection in ophthalmology)	- Good response - Enhance uptake the numbers of screening in short periods (3 months)	- Effective & efficient - Groups with incentives can work more	- Providing incentives to ASHAs was of no extra advantage. While incentives could increase screening uptake, it was more selective, particularly in people with uncontrolled diabetes, low literacy, and a longer duration of diabetes than the HE-only phase - Cadres can be used effectively to refer known PwD for DR screening especially when DR screening program is introduced in population with low awareness and poor accessibility to increase uptake of DR screening

*ASHA* Accredited social health activist, *HE* Health education, *DR* Diabetic retinopathy, *PwD* People with Diabetes

#### Challenges

The first and foremost challenge found in studies is inadequate infrastructure. First, access to primary health care is difficult and expensive for people living in rural areas [11, 34]. For example, in India in Maharashtra district regions far from primary care health facilities [11]. They will attend the facility if they are facilitated. As mentioned by Singh et al., their study revealed that the uptake was significantly higher to PHC facilities because of the provision of transport to PHC from villages [11]. Therefore, delivering care closer to the people is equally important. This study shows more acceptance for DR screening in the PHC closer to the residence. However, this is possible only with an adequate increase in infrastructure and skilled workforce [11, 12]. Besides, in rural areas, health workers are sometimes not provided with adequate equipment, such as outdated, incomplete, and limited diagnostic tools and medicines, and the distance of health facilities is far. In contrast, in city and suburban areas, health equipment is quite complete, medicines can be easily found, and the distance of health facilities is very close [11, 35]. Lastly, awareness regarding the disease and its risk is low due to the scarcity of literature [11, 12, 34]. Most people believe that a screening examination is only essential when eye problems appear [33]. Patients are frequently identified with DR in a severe, vision-threatening stage, making treatment challenging [3, 5].

In addition, another challenge found in the studies is that the process originates from patients and cadres themselves [26]. First, the high poverty rates in rural areas result in people being unable to access health care because they do not have enough money for transportation, which is sometimes far away and quite expensive [12, 33, 34]. Second, the lack of awareness within the communities made the screening process difficult. Third, the trust issue in society towards cadre and cadre towards local medical health workers aggravates the health care process. For instance, when cadres want to promote health, the community distrust’s identity cadres because non-medical workers require them to show their identity. Next, the cadre’s problem with other health workers in primary health care (nurses) who canceled meetings with the community suddenly resulted in community disappointment [33]. Last, the educational attainment issue in LMICs became central to this vicious cycle of health problems. A recent survey study revealed that most people of productive age (15–49 years old) had not completed primary education [36]. Consequently, this problem also affected the language barrier, which limited the community's ability to understand health promotion and the importance of eye examinations [33, 36]. A summary of the challenges to the role of cadres is summarized in Table 4.

**Table 4:** The challenges

No	Author	Year	Country	Challenges
1	Rani et al	2005	India	NA
2	Singh S et al	2020	India	Lack of awareness, accessibility, affordability, poor infrastructure, lack of skilled manpower and outdate technology
3	Mwangi et al	2020	Kenya	- Lack of referral case from diabetes services (low referral case) - Lack of knowledge of diabetes eye complications among PwD, and the belief that a screening exam is only necessary once ocular symptoms develop (Lack of awareness)
4	Ram S et al	2022	Fiji	Major Challenges: 1. People living in poverty cannot afford a balanced diet, transportation costs to a health facility, and other expenses involved in caring for a patient. (poverty) 2. People faced long waiting times at health facilities, which was frustrating for elderly patients who travelled long distances, were unaccompanied and were poor (infrastructure & services) 3. Some individuals did not acknowledge the services rendered by the Cadre and were not cooperative, and needed the Cadre to present their identity cards (trust issue) 4. Sometimes scheduled community outreach screenings were cancelled at the last minute by the nurses (trust issue) Other challenges: 1. Patients being late or not presenting to a health facility despite being advised by the CHWs on adverse health outcomes (Lack of awareness) 2. Cadre not being bilingual to disseminate health messages effectively a (cadre's barrier) 3. Lack of dedicated spaces in settlements for screening during outreach and confidential patient counseling, compared to dedicated village/communal halls in traditional Fijian villages (infrastructure) 4. Cadres difficulty in putting together a community profile and forgetting the knowledge gained over time (cadre's barrier) 5. Cadres are not supplied with resources and consumables (Lack of service from local government)
5	Shah et al	2018	Pakistan	NA
6	Chariwala et al	2020	India	- Monetary incentives are not sustainable; in the short run, it may be beneficial but in the long run it needs to be phased out and education and self-care by nonincentivized mechanism should drive PwD for DR screening

*CHWs* Community health workers, *PwD* People with Diabetes

## Discussion

DR can lead to potentially blinding problems, which can be avoided by early detection with routine dilated fundus checks and referral as necessary [37]. In diabetic care facilities, the necessity of early diagnosis and screening is highlighted [38]. The screening target for DR cases is expected to reach 80% in 2030 [7, 8]. It is challenging for the government, ophthalmologists, and eye care providers to carry out their societal duties. Previous studies have identified several barriers to implementing good DR screening, including the lack of ophthalmologists, uneven distribution of ophthalmologists, lack of awareness in society, accessibility and affordability of health care facilities, poor infrastructure, a lack of skilled workforce, and outdated technology [12, 34, 39]. Indeed, due to the uneven distribution of ophthalmologists, most ophthalmologists have been overwhelmed with a rapidly growing population of diabetics, making it impossible to expect them to examine the whole population. As a result, ophthalmologists require assistance from community members in achieving targeted goals, particularly in rural areas.

The role of cadres in Indonesia has been recognized since 2019 through a law issued through the regulation of the Minister of Health of the Republic of Indonesia number 8 in 2019 regarding community development in the health sector, where a cadre is anyone who is chosen by the community and trained to mobilize the community to participate in community empowerment in the health sector [40]. Cadres play a role in improving the knowledge and ability of the community to recognize and address health problems. These community services include maternal health, infants, school children, productive age, the elderly, community nutrition, communicable and non-communicable diseases, and mental health [40]. Based on these functions and roles, task sharing is a solution to the growing shortage of eye care personnel to manage eye care services for patients with diabetes [17]. The WHO defines task shifting as the transfer of tasks to existing cadres of healthcare professionals with less training and credentials or to newly developed cadres who obtain competency-based training for the specific activity [18]. As a result, the objective of universal coverage can be reached with this task-shifting method provided the screening is cost-effective, covers the target group, and is accepted by the community, particularly in the context of controlling blindness due to DR [41, 42].

Furthermore, one of the possible areas for task sharing or shifting is health education, and available cadres might be assigned new duties related to DR education and awareness. Shah et al. found the importance of cadre and diabetes educators in educating people with diabetes about DR and associated risk factors in Pakistan [17]. Similarly, they can help detect high-risk diabetics as part of their regular house-to-house visits for maternal and child health care [17]. The recommendations of various cadres for health education were similar to the role of cadre in mother and child health education in Pakistan, [43, 44] as well as the Aravind Eye Hospital and the LV Prasad Eye Institute models, where health education is provided by trained community health workers chosen from the same community [45, 46]. Therefore, work sharing should not only be considered a solution for the ophthalmologist shortage but also part of an entire strategy in a successful health system. Another study by Singh that emphasized the role of cadres (ASHAs) revealed that ASHA participation in giving health education to people with diabetes could enhance DR screening [11]. ASHAs may share tasks as local change agents, role models, and mentors [17]. Performing DR screening closer to the health facilities with transportation and health education was more successful, resulting in increased DR screening uptake among patients known to have diabetes in remote areas [11, 17].

In India, the ASHA is the cadre that serves as a connection between the community and the health system, as agents of social change for health promotion, and as the primary pillar of achieving government policy goals at the grassroots level [11]. DR is the sixth-leading cause of blindness and visual impairment in India [47]. Due to the scale of the problem in India, DR screening gains excellent relevance. This service must be simple and low-cost to be effective in a resource-constrained nation like India. According to the Diabetic Retinopathy Study and the Early Treatment Diabetic Retinopathy Study, laser photocoagulation can minimize 90% of severe vision loss when administered on time [48, 49]. Therefore, ASHA might bridge this knowledge gap and work as a motivator to encourage more patients to seek DR screening at the primary care level at Community Health Centers [50, 51]. For the same reason, this can be a reference for Indonesia, where DR is also the fifth cause of blindness [14], so the cost-effective screening system can be learned and applied in Indonesia. In addition, recruiting cadres (ASHA) in India is inspiring and worth remembering.

Besides having a cost-effective screening system, the ASHA model in India also provides an effective and efficient model. First, a cadre from India is part of the community. Cadres in India are women village residents who are married, widowed, or divorced, preferably between the ages of 25 and 45. They are chosen and trained to mediate between the community and the public health system. Second, the selection process is tight and serious. The ASHA will be chosen through a rigorous selection process and will get 23 training days divided into five episodes. Asha's training is held continuously to equip them with essential skills and competence through on-the-job training [52, 53]. Third, the ASHA works to be educated with DR screening and knowledge and other useful understanding regarding the frequently happening health issues**.** Following six months of working in the community, the ASHA will receive several competencies including HIV/AIDS topics such as sexually transmitted infections, respiratory tract infections, prevention, referrals, and newborn care. It is what characterizes cadres in India since they are authorized under government laws and procedures [27]. Lastly, ASHA has enough knowledge of the villager's health information. After selecting ASHAs, the next phase would familiarize her with the villagers' health status and help her adapt to rural settings. Even though ASHA is from the same village, she may not be aware of or have information about the village's current health. Towards that purpose, she should be advised to visit every family and conduct a sample survey of the village's population to ascertain their health [50, 52]. This way, she will have the opportunity to know the villagers, the common diseases that impact them, the number of pregnant women, the number of newborns, the educational and socioeconomic status of various groups of people, the health status of the weaker sections, particularly scheduled castes and scheduled tribes, and so on. They also can be given a basic structure for doing surveys [27, 50, 52].

A study by Shukla et al. evaluated a training program to engage ASHAs in delivering primary eye care to vulnerable urban populations [51]. The ASHAs will give training about the basic structure and function of eyes through an eye model. They knew the definitions of blindness and visual impairment and their causes. ASHAs were given an overview of common eye conditions and their referrals [51]. Afterward, ASHAs were given hands-on training in screening the vision of individuals aged ≥ 40 using two "E" charts of 6/60 and 6/18 optotypes. They provided a training kit comprising measuring tape, screening cards, referral slips, and educational material. They were given 3–4 months to screen the vision. Apart from vision screening, ASHAs could indicate in referral slips if the person had diabetes, diagnosed glaucoma, symptoms of presbyopia (near vision difficulties after 40 years of age), or any other eye conditions [51]. As a result, ASHAs showed a significant increase in knowledge immediately after training, sustained even after a year. They enhanced their knowledge about common eye diseases such as cataracts, glaucoma, the effects of diabetes on the eyes, presbyopia, and conjunctivitis, and they would be able to talk confidently in the community about eye care. Then, the ASHA referral increased by more than four times [51].

In conclusion, investing in scalable approaches such as Cadre training is a critical first step in managing diabetes and DR in communities, particularly at the grassroots level in low-resource settings, by improving community awareness of DR and improving access to screening, diagnosis, and treatment [33].

Furthermore, a good-quality cadre will have an impact on society. People are motivated to attend the screening event because of the cadre’s motivation. Mwangi et al. revealed that eighty percent of members attend at least two-thirds of the meeting annually. In addition, the uptake numbers for screening were increased to achieve effective DR screening [32]. Rani et al. claimed that they could detect new cases of diabetes mellitus out of the 4.5 percent that might have a risk for DR, and community participation is the key to success for any awareness or screening model [16].

In addition to increasing awareness, cadres help to reach a more cost-effective screening process. It is cost-effective because there is potential to increase screening uptake in a relatively short period (3 months), with a striking uptake in the first two weeks [11]. The rate of incidence of eye examination was about six times higher compared to the control group before the start of the intervention compared to control group.. They could begin referring not only the existing diabetes patients with vision loss or blindness for DR screening but also those who were 40 years of age or older and had other symptoms such as vision loss, blindness, or increased thirst to visit a health facility for screening for diabetes [33]. In contrast, providing incentives to ASHAs was of no extra advantage. While incentives could increase screening uptake, they were more selective, particularly in people with uncontrolled diabetes, low literacy, and a longer duration of diabetes than in only education groups [31].

Cadres also can be used effectively to refer PwD for DR screening, especially when a DR screening program is introduced in a population with low awareness and poor accessibility to increase uptake of DR screening. This good response of society is assisted by the cadre's ability to speak the local language so that the community can more easily understand and understand the invitation and motivation of the cadre [33]. Cadre could be a good supporter and an excellent reminder to society as they gain respect and acknowledgment for their work, resulting in a positive response from the community [32, 33]. It becomes a continuous positive root formed in the community, and cadres feel like an essential part of the health system. They created a link between the community and diabetes healthcare professionals.

The role of good cadres in the community is also not free from challenges in the field. As mentioned, there are several challenges in providing eye health services to reduce blindness due to DR. In addition to the unbalanced distribution of ophthalmologists and the overworked capacity of doctors to meet targets, other issues also affect this community-based health service. First, accessibility to primary health care remains difficult and unaffordable for people in rural areas [11]. Second, the infrastructure to support diagnostics and services is outdated compared to healthcare facilities in urban areas. As a result, the number of DR referrals seems low or goes unreported due to barriers in diagnosing and accessing referrals [32]. Third, awareness of the disease is low due to the scarcity of literature and understanding of the importance of early screening to prevent complications due to diabetes. Society believes that a screening exam is only necessary once ocular symptoms develop [32]. Consequently, people are often diagnosed with DR in a severe, sight-threatening phase, making management difficult [3, 5].

Furthermore, within the communes there are some challenges the society is dealing with. According to Ram S et al., is that people living in poverty cannot afford a balanced diet for a healthy life to prevent diabetes. Transportation costs to health facilities and other expenses required to treat patients are expensive and unaffordable for the community [33]. Sometimes, if they could attend a health facility, they would face long waiting times, frustrating for elderly patients who travel long distances [12]. This is compounded by the challenges faced by cadre when they are doing their task in the communities; the individuals are having relatively trust issues regarding the cadre's role as a medical helper or questioning their role so that the cadre must show his identity in front of the community. The language barrier of some cadres, who do not speak the local language, limits the community's ability to understand the context of health promotion and the importance of eye examinations [12, 33]. Consequently, people are sometimes late or do not present themselves at a health facility despite being advised to do so by the cadre, even though they have been explained the dangers of the disease. When the community manages to come to PHC to receive counseling and basic examinations by health workers, sometimes it must be canceled at the last minute by the nurse so that it becomes a matter of trust by the community because they are disappointed that their presence is a waste of time [12, 33]. Some of these problems have emerged in the field and are experienced by cadres and the community.

In addition, government supervision and appreciation of cadres are not adequate. Many cadres who serve in the community are not given proper incentives and resources to fulfill their role in the community [12, 31]. In India, despite the cadre's excellent training and recruitment process, they receive small and irregular incentives, which can demotivate ASHAs. However, Chariwala said that sometimes incentivizing cadres based on how much they can afford to spend on health education, health promotion, and motivating people to get checked, come to PHC facilities, and get treated at secondary and tertiary health services is not sustainable [31]. The provision of incentives may be helpful in the short term, but in the long run, it needs to be phased out, and education and self-care through non-incentivized mechanisms should drive PwD for DR screening [31, 51].

### Strengths

This is the first scoping review to explore the role of cadre in the community in DR management and its challenges in LMICs. This review has identified gaps in the existing literature regarding the role of the cadre in the management of DR and its challenges in LMICs setting, which is particularly important as it highlights the role of the cadre and its barriers to reducing blindness due to DR through community-based intervention. This study successfully explored task shifting by cadres in DR management. Then, cadres are also known to create relationships between the community and diabetes health professionals. In this study, we also evaluated the outcomes of the cadre’s efforts to uptake diabetic eye screening, which will increase referrals and is also known to create relationships between the community and diabetes health professionals. After that, this study also explains the community's behavior towards cadres. In addition, this study also describes the barriers that occur in the community, not only from the perspective of the community but also from the perspective of the cadres themselves. The findings in this study are useful for program planners and policymakers to implement task shifting by healthcare providers in their respective countries.

### Limitations

The lack of good-quality publications regarding the cadre's role, focusing on DR in LMICs, was a significant challenge in this scoping review. Despite the author's best efforts, relevant evidence may inevitably have been missed. After all, it is difficult to get a good-quality journal based on the Scopus standard because most are paid journals. Then, the variety of term cadres creates obstacles in this scoping review. The author must do a full-text review which takes time.

### Implications and recommendations

This study is a potential recommendation for LMICs as a model for implementing a task-sharing system carried out by non-health workers as a guideline for community-based interventions. The DR screening program in each country could be maximized by looking at the mapping study from this research. This community-based intervention can also reactivate the healthcare pyramid so that universal coverage is expected to be achieved. The recruitment, training, and handling of cadres can be learned from this study to involve cadres in managing DR. In this study, the role of cadres had a good response in the community. The community's enthusiasm for eye screening, which resulted in a high referral coverage rate, could be one way to achieve the DR screening coverage target of 80% by 2030.

Nevertheless, the role of this cadre should not be forgotten by the government and health authorities in the processes of supervision, training, protection, and welfare. These need to be outlined in detail and with clarity in law and policy related to the operational standards of the work of the cadre itself. Therefore, the role of cadres here is expected to be not only to help health workers but also to act as a bridge between the community, health workers, and the government to reduce the country's blindness rate.

## Conclusion

The current study highlighted significant gaps in the literature that focus on the cadre's role as a community-based intervention in managing DR in LMICs. From this study, we can find that cadre can motivate people to come to the diabetic eye screening event. The rate of incidence of eye examaminations was about six times higher compared to the control group before the start of the intervention. They could begin referring not only the existing diabetes patients with vision loss or blindness for DR screening but also those who were 40 years of age or older and had other symptoms such as vision loss, blindness, or increased thirst to visit a health facility for screening for diabetes [33]. Education is a possible area for task sharing. Moreover, 70% reported that the cadre could perform the task of vision testing [17]. Additionally, the cadre could be a good peer supporter and an excellent reminder to society as they gain respect and acknowledgment for their work, resulting in a positive response from the community. Hence, developing a targeted research agenda based on recent results is a crucial next phase in generating the essential improvements within and across LMICs to address the existing and upcoming issues of DM and DR. Further research is needed to develop a body of evidence that is adequate to support cost-effective screening services and cadre-related policy development in LMICs. As a result, the national government and district governments should take a leadership role in developing and implementing comprehensive policies that make DR prevention a national policy.

### Supplementary Information

**Additional file 1.** Search terms in each database. 

**Additional file 2.** Operational definition.

## Data Availability

All data generated or analyzed during this study are included in this published article and its supplementary information files.

## Abbreviations

ASHA: Accredited social health activist
CHC: Community health centers
CHWs: Community health workers
DM: Diabetes mellitus
DR: Diabetic retinopathy
DSG: Diabetes support groups
IDF: International diabetes federation
IEC: Information, education, and communication
LHW: Lady health worker
LMICs: Low- and middle-income countries
PCC: Population, concept, context
PHC: Primary health care
PwD: People with diabetes
RCT: Randomized controlled trial
VHW: Village health workers
