# Supplementary material for: The role of cadre in the community on diabetic retinopathy management and its challenges in low-middle income countries: a scoping review
Source: BMC Public Health. 2024 Jan 15;24:177. doi: 10.1186/s12889-024-17652-5 (PMC10789068; doi:10.1186/s12889-024-17652-5)
Supplement: Supplementary file 1 — Additional file 1. Search terms in each database. [file 12889_2024_17652_MOESM1_ESM.docx]

Additional file 1 Search terms in each database

| **Database** | **Search terms (MESH or keyword)** |
| --- | --- |
| Cochrane (1399) | (Cadre) AND (Diabetic Retinopathy), (Cadre) AND (Diabetic Retinopathy) AND (Screening), (Cadre) AND (Screening Diabetic Retinopathy) AND (Management), (Community health worker) AND (Screening Diabetic Retinopathy), (Diabetic eye disease or diabetic retinopathy) AND (adult* not (pediatric* or adoles* or child* or infan*)) in Title Abstract Keyword AND (cadre or community health worker*) in Title Abstract Keyword AND (Afghanistan or Burkina Faso or Burundi or Central African Republic or Chad or Democrati? Republi? of the Congo or Eritrea or Ethiopia or The Gambia or Guinea or Guinea-Bissau or Democratic People's Republic of Korea or Liberia or Madagascar or Malawi or Mali or Mozambique or Niger or Rwanda or Sierra Leone or Somalia or South Sudan or Sudan or Syrian Arab Republic or Togo or Uganda or Republican Guard or Yemen or Zambia or Angola or Algeria or Bangladesh or Benin or Bhutan or Bolivia or Cabo Verde or Cambodia or Cameroon or Comoros or Republic of the Congo or Republic of Cote d'Ivoire or Djibouti or Egypt or The United Arab Republic or El Salvador or Eswatini or Ghana or Haiti or Honduras or India or Indonesia or Islamic Republic of Iran or Kenya or Kiribati or Kyrgyz Republic or Lao PDR or Lebanon or Lesotho or Mauritania or Federated States of Micronesia or Mongolia or Morocco or Myanmar or Nepal or Nicaragua or Nigeria or Pakistan or Papua New Guinea or Philippines or Samoa or (Sao Tome and Principe) or Senegal or Solomon Islands or Sri Lanka or Tanzania or Tajikistan or Timor-Leste or Tunisia or Ukraine or Uzbekistan or Vanuatu or Vietnam or (West Bank and Gaza) or Zimbabwe) or ((low and middle income countr*) or (low and middle income econom*) or developing countr* or OECD countr*) in All Text |
| Pubmed (998) | (Cadre) AND (Diabetic Retinopathy), (Cadre) AND (Diabetic Retinopathy) AND (Screening), (Cadre) AND (Screening Diabetic Retinopathy) AND (Management), (Community health worker) AND (Screening Diabetic Retinopathy), (Diabetic eye disease or diabetic retinopathy) AND (adult* not (pediatric* or adoles* or child* or infan*)) in Title Abstract Keyword AND (cadre or community health worker*) in Title Abstract Keyword AND (Afghanistan or Burkina Faso or Burundi or Central African Republic or Chad or Democrati? Republi? of the Congo or Eritrea or Ethiopia or The Gambia or Guinea or Guinea-Bissau or Democratic People's Republic of Korea or Liberia or Madagascar or Malawi or Mali or Mozambique or Niger or Rwanda or Sierra Leone or Somalia or South Sudan or Sudan or Syrian Arab Republic or Togo or Uganda or Republican Guard or Yemen or Zambia or Angola or Algeria or Bangladesh or Benin or Bhutan or Bolivia or Cabo Verde or Cambodia or Cameroon or Comoros or Republic of the Congo or Republic of Cote d'Ivoire or Djibouti or Egypt or The United Arab Republic or El Salvador or Eswatini or Ghana or Haiti or Honduras or India or Indonesia or Islamic Republic of Iran or Kenya or Kiribati or Kyrgyz Republic or Lao PDR or Lebanon or Lesotho or Mauritania or Federated States of Micronesia or Mongolia or Morocco or Myanmar or Nepal or Nicaragua or Nigeria or Pakistan or Papua New Guinea or Philippines or Samoa or (Sao Tome and Principe) or Senegal or Solomon Islands or Sri Lanka or Tanzania or Tajikistan or Timor-Leste or Tunisia or Ukraine or Uzbekistan or Vanuatu or Vietnam or (West Bank and Gaza) or Zimbabwe) or ((low and middle income countr*) or (low and middle income econom*) or developing countr*) in All Text |
| Embase (380) | (Cadre) AND (Diabetic Retinopathy), (Cadre) AND (Diabetic Retinopathy) AND (Screening), (Cadre) AND (Screening Diabetic Retinopathy) AND (Management), (Community health worker) AND (Screening Diabetic Retinopathy), |
