# Supplementary material for: The role of cadre in the community on diabetic retinopathy management and its challenges in low-middle income countries: a scoping review
Source: BMC Public Health. 2024 Jan 15;24:177. doi: 10.1186/s12889-024-17652-5 (PMC10789068; doi:10.1186/s12889-024-17652-5)
Supplement: Supplementary file 2 — Additional file 2. Operational definition. [file 12889_2024_17652_MOESM2_ESM.docx]

Additional file 2 Operational Definition

| **Terms** | **Definition** |
| --- | --- |
| Diabetic retinopathy (DR) | Ocular manifestation of end-organ damage in diabetes mellitus which affects the microvascular of the retina^[19]^ |
| People with Diabetes (PwD) | All persons with newly diagnosed (provisional) diabetes, plus persons with known diabetes (referred from general physicians and diabetologists)^[20]^ |
| Cadre | Healthcare workers with shorter training and fewer qualifications or newly created cadres who receive competency-based training for the specific task^[21]^ |
| DR Screening | Assessment of the target population for the presence of DR through fundus examination in a pupil dilatation condition by using an ophthalmoscope of direct/indirect/slit lamp examination + 75 or 90 D lens/retina photography/oct (to examine macular edema) by an individual who is competent to perform it.^[22]^ |
| Health education | Health education is an approach to delivering targeted health promotion and illness prevention programs. Health education offers targeted audiences with knowledge on specific health subjects, including health benefits and risks, as well as methods to build capacity and promote behavior change in an appropriate setting.^[23]^ |
| Low Middle-Income Countries (LMICs) | Classification of countries based on their income according to the world bank criteria^[6]^ |
| Referral Systems in Indonesia | Referral system occurs on a hierarchical basis from primary (being the nearest health facilities to the community such as Puskesmas, private clinics, type D hospitals), secondary (Type B Hospital), and tertiary health facilities (Type A Hospital).^[24]^ |
| Primary health care/public health care/community health care | First-level health facility^[24]^ |
